# Bridging perception and reality: Parasitic identification in delusional parasitosis

**DOI:** 10.1371/journal.pone.0358452

**Published:** 2026-09-18

**Authors:** Tippayarat Yoonuan, Watcharapong Piyaphanee, Sompan Srinukham, Ubolwan Wonguten, Prakaykaew Charunwatthana, Dorn Watthanakulpanich

**Affiliations:** 1 Department of Helminthology, Faculty of Tropical Medicine, Mahidol University, Bangkok, Thailand; 2 Department of Clinical Tropical Medicine, Faculty of Tropical Medicine, Mahidol University, Bangkok, Thailand; 3 Hospital for Tropical Diseases, Faculty of Tropical Medicine, Mahidol University, Bangkok, Thailand; Niigata University of Pharmacy and Medical and Life Sciences, JAPAN

## Abstract

**Background:**

Delusional parasitosis (DP) is a psychiatric condition in which individuals hold a fixed, false belief that they are infected with parasites. These patients frequently consult parasite clinics and submit household or biological debris, often nonparasitic in origin, for parasitological examination. Although parasitologists are not directly responsible for treating this psychiatric disorder, they play a key role in identifying or excluding parasitic infections and guiding appropriate care. Misidentification of artifacts as parasites may result in unnecessary treatment, prolonged distress, and, in some cases, delayed diagnosis of DP. Thus, this study examined specimens submitted for parasitological diagnosis with the aim of assessing diagnostic accuracy and evaluating the role of laboratory confirmation in managing suspected parasitic infections.

**Methods:**

This retrospective study analyzed 382 clinical specimens submitted for parasitological examination to the Department of Helminthology, Faculty of Tropical Medicine, Mahidol University (June 2014–June 2022). All specimens were examined macroscopically and microscopically using standard parasitological techniques.

**Results:**

Parasitic infections were present in 108 (28.3%) specimens, including nematodes, cestodes, and trematodes, such as *Trichuris trichiura*, *Enterobius vermicularis*, hookworm, and *Taenia* spp. The remaining 274 (71.7%) specimens included negative fecal specimens and specimens comprising various artifacts, such as food residues, plant materials, synthetic fibers, skin flakes, and other debris. This study was concurrently performed using outpatient data obtained from the Parasite Excellence Clinic, Hospital for Tropical Diseases, Faculty of Tropical Medicine, Mahidol University. Notably, 84 patients were ultimately diagnosed with DP.

**Conclusions:**

Accurate parasite identification is crucial to ensure proper treatment in true parasitic infections and prevent DP misdiagnosis. This study highlights the importance of an integrated parasitology–psychiatry approach, particularly when patients present with persistent symptoms despite negative laboratory test results. These findings support the establishment of diagnostic protocols and improved interdisciplinary communication to enhance care for patients with suspected parasitic infections.

## Introduction

The increasing accessibility of health information online has led many patients to self-diagnose based on symptoms. Although the availability of information empowers patients, it also creates diagnostic challenges when expectations are unmet by laboratory test results. Patients often attribute unexplained symptoms to parasitic infections and seek laboratory test confirmation at specialized centers. However, despite repeated negative test results, some patients persist in their beliefs, raising suspicion of DP (DP; also known as Ekbom syndrome) [1]. DP is defined as a fixed false belief of infection in the absence of objective evidence, such as parasites, insects, vermin, or maggots, that persists for at least 1 month [2–5]. It is also referred to as dermatophobia, parasitophobic neurodermatitis, parasitophobia, or entomophobia [6,7]. DP is frequently associated with tactile and olfactory hallucinations, anxiety, and self-inflicted lesions [8]. However, clinical functioning is not markedly impaired, and behavior and personality show no apparent deterioration [9]. Furthermore, DP does not result from the direct physiological effects of a substance (e.g., a drug of abuse or a medication) or a general medical condition. There are two types of DP: primary DP, which is idiopathic, and secondary DP, which is either functional (associated with schizophrenia, paranoia, depression, and anxiety disorders) or organic (due to drug abuse, hypothyroidism, cancer, cerebrovascular disease, tuberculosis, neurologic disorders, vitamin B12 deficiency, or diabetes mellitus) [10–12]. Thus, accurate laboratory diagnosis is critical, not only for confirming true parasitic infections but also for ruling them out, which is essential for appropriately managing suspected DP [10,13]. Although psychiatric in origin, DP frequently presents in parasite clinics in Thailand, likely due to public perception, accessible parasitological services, and prevailing cultural beliefs.

This study investigates the types of specimens submitted for parasitological analysis, the outcomes of those examinations, and how laboratory test results influence clinical management. Special attention is given to persistently negative cases, where DP should be considered as a diagnosis. The present study aimed to evaluate the role of parasitological examination and morphological identification in distinguishing true parasitic infections from pseudoparasitic or non-parasitic materials among patients with suspected parasitosis, particularly those later diagnosed with DP. In addition, this study sought to characterize the types of submitted specimens, patterns of specimen referral, and demographic characteristics of patients presenting with suspected parasitic infections at the Hospital for Tropical Diseases, Faculty of Tropical Medicine, Mahidol University, during an 8-year retrospective period.

## Materials and methods

### Sample collection and analysis

From June 2014 to June 2022, 382 clinical specimens were analyzed by the Department of Helminthology at Mahidol University, including 122 specimens from internal sources (Parasite Excellence Clinic and Inpatient Department, Hospital for Tropical Diseases, Faculty of Tropical Medicine, Mahidol University) and 260 specimens from external sources (private laboratories and other hospitals). Fecal samples were examined for helminth eggs using the Kato–Katz method and simple smear technique. *Strongyloides stercoralis* was detected using either agar plate or polyethylene tube culture techniques [14,15]. Nonfecal samples and foreign objects were examined using stereomicroscopy. Body fluid samples were centrifuged, followed by microscopy examination of the sediment.

### Microscopy and identification

Specimens submitted by patients were examined by experienced parasitologists from the Department of Helminthology, Faculty of Tropical Medicine, Mahidol University. Personnel involved in specimen identification had formal training in medical parasitology and extensive experience in the morphological identification of helminths and pseudo-parasitic materials encountered in clinical practice. Identification procedures were performed using standard parasitological techniques, including stereomicroscopy, light microscopy, together with established taxonomic keys and diagnostic references. All specimens were systematically accessed for characteristic parasitic morphology, including egg morphology (3S: size, shape, shell, 2C: color, content), as well as the presence of specific characteristics such as esophagus configuration, proglottids, larvae, suckers, spines, cephalic alae, cervical alae, and bursae.

Accurately morphological identification requires both technical expertise and rigorous quality control procedures. Ambiguous, unusual, or fragmented specimens were reviewed and cross-checked among experienced senior personnel to minimize diagnostic uncertainty. Representative specimens were photographically documented and archived as reference materials when appropriate, and routine comparison with established morphological criteria was performed. Continuous training in helminth morphology and diagnostic parasitology was also emphasized to maintain diagnostic competency. Particular attention was paid to differentiating true parasitic organisms from non-biological materials and environmental contaminants, including synthetic fibers, plant materials, arthropod larvae, and other pseudoparasitic objects that may resemble helminths macroscopically or under low magnification. These quality control measures were especially important in DP-related submissions, where misidentification could reinforce false beliefs, delay psychiatric intervention, or lead to unnecessary antiparasitic treatment.

### Patient data collection and sample inclusion

Patients’ demographic data (age and sex) and clinical characteristics were obtained from the Medical Record Department at the Hospital for Tropical Diseases, Faculty of Tropical Medicine, Mahidol University. Due to ethical considerations, these data are not publicly available. Data were accessed for research purposes between 13/06/2024 and 15/09/2024.

A formal prospective sample size calculation was not initially performed because this retrospective observational study employed a consecutive inclusion (total sampling) approach, whereby all eligible specimens submitted between June 2014 and June 2022 were included. However, to assess the adequacy of the analyzed sample, a post hoc estimation for prevalence studies was performed using the formula n = Z2P(1−P)/d2n = Z^2P(1-P)/d^2n = Z2P(1−P)/d2. Due to limited epidemiological data regarding delusional parasitosis-related specimen submissions, a conservative expected proportion of 50% was applied with a 95% confidence level and 5% precision. The estimated minimum sample size was 384 specimens, which was comparable to the final analyzed dataset of 382 specimens.

### Ethical considerations

This retrospective study used data from medical records. The study was approved by the Faculty of Tropical Medicine Ethics Committee, Mahidol University (MUTM 2024-047-01), approved on 12 June 2024). All data were fully anonymized before being accessed for research purposes.

## Results

### Outcomes of parasitological examination

Of the 382 analyzed specimens, 108 (28.3%) tested positive for parasites, cestodes and nematodes were the most frequently identified helminths including *Trichuris trichiura*, *Enterobius vermicularis*, *Hymenolepis nana*, and *Taenia* spp. which hookworms and *Taenia* spp. represented the largest proportion of submitted intact or partially worm specimens. Most positive identification were achieved through preserved characteristic morphology despite fragmentation or partial degradation of the submitted specimens. The remaining 274 (71.7%) specimens included negative fecal and body fluid specimens or specimens of nonparasitic materials, such as plant debris, undigested mushrooms, synthetic fibers, and nonparasitic organisms (Table 1).

**Table 1:** Type and number of specimens submitted to the Department of Helminthology, Mahidol University, for parasitological examination.

Specimens	Total	Positive	Not found/ Not parasite
Feces	161	10	151
Body Fluid	42	4	38
Partial Worm/ Worm	96	94	2^a^
Worm-like Material/ Insect Larvae	74	0	74
Tissue/ Skin Scarp	9	0	9
**Total**	**382**	**108**	**274**

^a^Two specimens were degraded and could not be identified.

**Fig 1:**
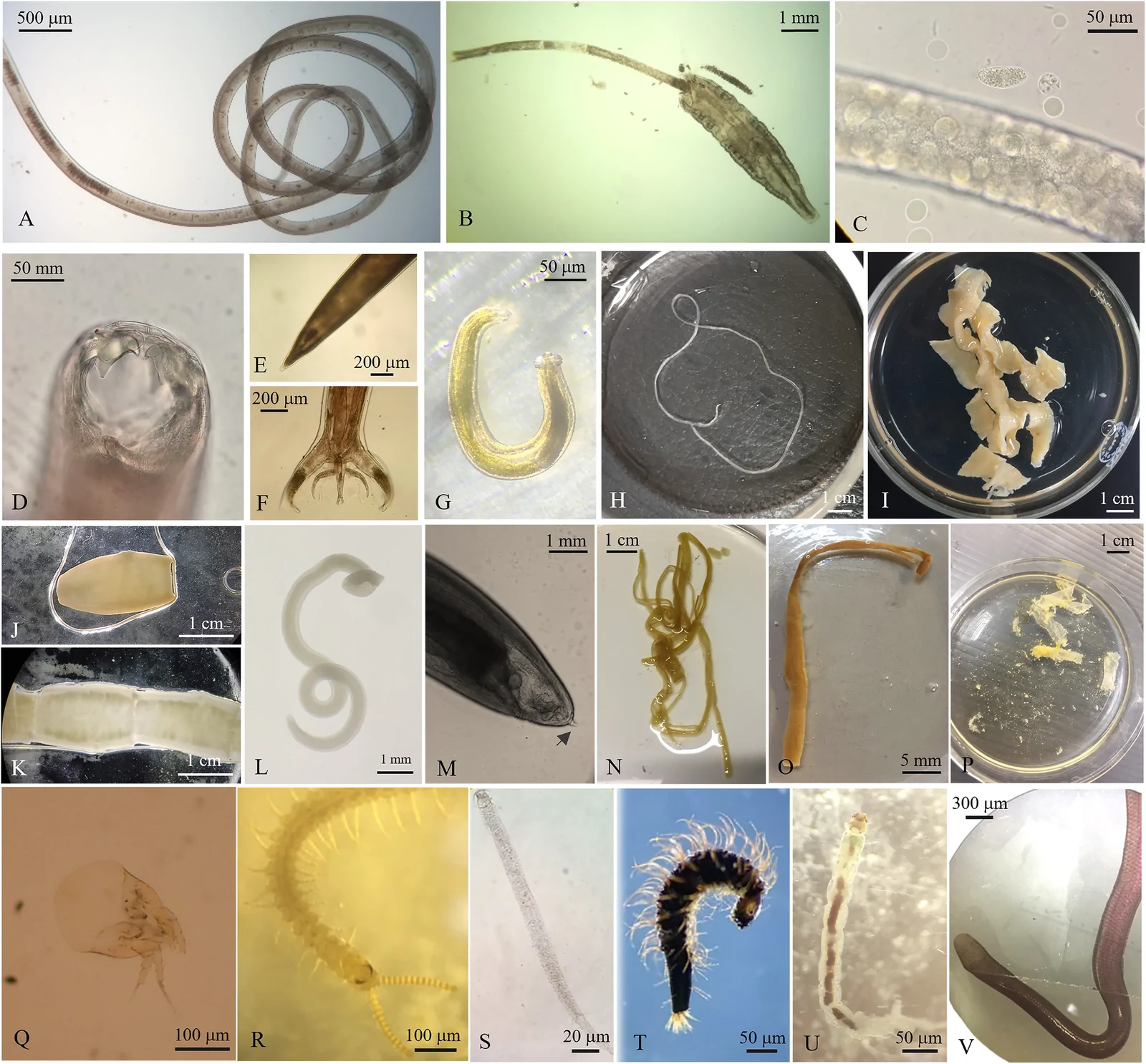
Specimens submitted to the laboratory. (A) partial worm specimen of *Trichuris* spp.; (B) partial worm specimens of *E. vermicularis*; (C) magnified detail of (B) showing a D-shaped egg of *E. vermicularis*; (D) buccal capsule of *Ancylostoma ceylanicum* showing teeth; (E) posterior end of female hookworm; (F) posterior end of male hookworm; (G) *Gnathostoma* larva; (H) *Dirofilaria* spp.; (I) short strobila fragment of *Diphyllobothrium latum*; and (J) segment of *Taenia* spp.; (K) gravid proglottid of *Taenia* *saginata* showing uterine branches; (L) *Anisakis* spp.; (M) showing the mucron of *Anisakis* spp.; (N–P) plant fragments (N-O, Enokitake mushroom (*Flammulina velutipes*)); (Q–U) non-helminthic organisms, and (V) a common blind snake (*Indotyphlops braminus*).

Among the positive cases, all specimens containing worms, some of which only consisted of fragments, were carefully examined and identified based on specific morphological characteristics. Among the 35 partial worm specimens, certain morphological characteristics remained preserved, facilitating identification. One specimen contained creamy-colored fragments that were long, thin, and thread-like, with a width < 0.5 mm. Microscopic examination revealed a stichosomal esophagus. Although the posterior end of the worm was absent, these features were sufficient to identify the specimen as *Trichuris* spp. (Fig 1A). Another specimen was identified as *E. vermicularis*. Although it was incomplete, approximately one-third of the body was present, and microscopic examination revealed a portion of the uterus containing numerous D-shaped eggs, characteristic of *E. vermicularis* (Fig 1B and 1C). Both male and female specimens of the zoonotic hookworm *Ancylostoma ceylanicum* were identified (Fig 1D–1F). Other specimens identified based on preserved distinct morphological features included *Gnathostoma* larvae (Fig 1G), *Dirofilaria* spp. (Fig 1H), *Diphyllobothrium latum* (Fig 1I), and *Taenia* spp. (Fig 1J, 1K). One specimen consisted of a tiny strobila, ~1 cm long, composed of gravid proglottids, each <1 mm wide. The segments contained colorless eggs measuring 30–40 µm in diameter, with an oncosphere and characteristic polar filaments. These features were consistent with *H. nana*. Although *Anisakis* spp. is an uncommon parasite in Thailand, four larval specimens from a Japanese patient were submitted by an external laboratory (Fig 1L and 1M). Among the 161 fecal samples submitted, 10 (6.21%) contained helminthic eggs.

The most commonly submitted specimens were nonhelminthic foreign objects. Several specimens contained undigested plant fibers (Fig 1N–1P), such as the Enokitake mushroom (*Flammulina velutipes*, also known as the golden needle mushroom). When consumed, Enokitake mushrooms are not always fully digested, resulting in the excretion of visible, long, slender stalks and small caps in the feces. Other nonhelminthic specimens included larvae collected from toilet water. These larvae typically exhibited segmented bodies, sometimes with pairs of prolegs or legs, as well as anal setae and antennae. Vermiform larvae, such as midge larvae from the order Diptera, were also observed. Other samples resembling worms were identified in specimens collected in bathrooms or flush toilets (Fig 1Q–1U). There were a few cases of juvenile earthworms. One live, worm-like specimen, dark brown in color and showing active movement, was received with a note requesting identification to determine whether it was an *Ascaris* worm. Mouthpart examination revealed the absence of three lips. The specimen had two tiny black eye-like dots and scaled skin, identifying it as a common blind snake (*Indotyphlops braminus*) (Fig 1V).

**Fig 2:**
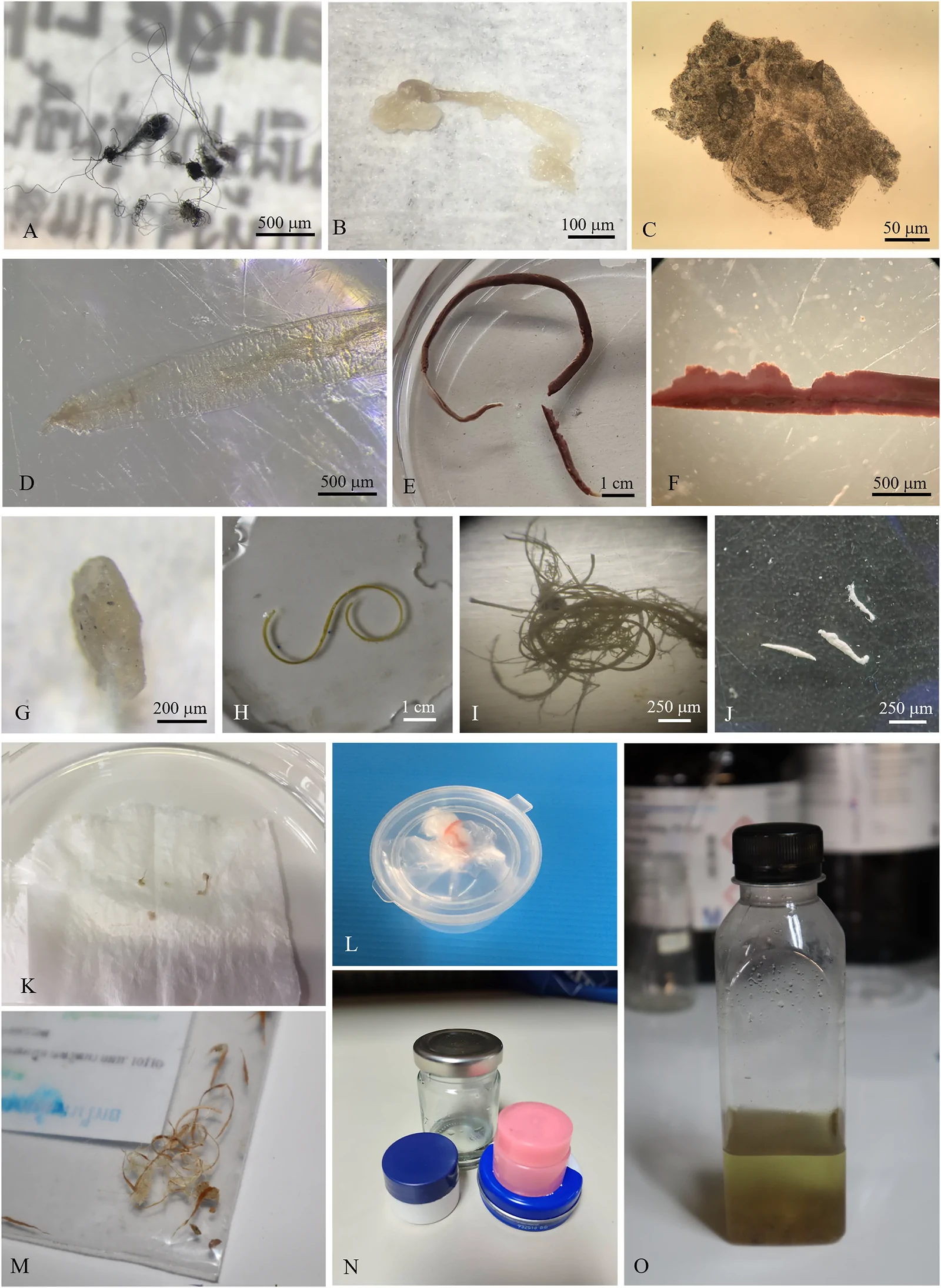
Other nonhelminthic submissions from patients diagnosed with delusional parasitosis. (A) fiber; (B-D) crusted discharge; (E-G) unidentified objects, (F, magnified detail of E); (H-I) plant fiber (I, loofah); (J) pieces of roll wet tissue; and (K-O) containers.

Other nonhelminthic submissions included synthetic fibers (Fig 2A), crusted discharge (Fig 2B–2D), unidentified objects (Fig 2E–2G), plant fiber (Fig 2H), loofah fragments (Fig 2I) and small rolls of wet tissue paper (Fig 2J). These items were often submitted wrapped in tissue paper (Fig 2K) and placed in household containers, including cosmetic jars, drinking water bottles, and ziplock plastic bags (Fig 2L–2O).

### Temporal trends and referral patterns

Although 108 specimens (28.3%) were confirmed to contain parasites, most submissions (274/382, 71.7%) consisted of non-parasitic materials, degraded specimens, environmental contaminants, or specimens in which no parasites were detected. These findings indicate that most submitted materials represented pseudoparasitic or nonhelminthic objects rather than true parasitic infections. The pattern of specimen submissions also reflected increasing referrals of patients with persistent concerns regarding parasitic infections, particularly among individuals later diagnosed with DP. Many patients repeatedly sought parasitological confirmation despite previous negative investigations and submitted a wide variety of materials collected from feces, skin, bathrooms, household environments, and personal belongings. Some patients attended the clinic on several occasions or submitted recurrent specimens through external hospitals and laboratories before a final diagnosis of DP was established. These repeated consultations and persistent parasitic concerns illustrated the prolonged diagnostic trajectory commonly encountered in DP patients and highlighted the challenges in distinguishing true parasitic infection from delusional infestation in routine clinical practice.

Distinct differences were observed between specimens submitted by DP patients and those from patients with confirmed parasitic infections or transient non-DP concerns. Confirmed parasitic infections most commonly involved intact worms, partial worm fragments, fecal specimens containing eggs, or other clinically relevant biological samples. In contrast, DP patients more frequently submitted non-biological or environmental materials, including synthetic fibers, plant debris, tissue fragments, crusted discharge, wet tissue paper, insect larvae, and household contaminants. Synthetic fibers and textile-like materials were particularly common and were often perceived by patients as motile or invasive organisms. Plant fibers and undigested food residues, especially mushroom fragments, also represented recurrent source of misidentification.

The relatively low proportion of positive findings among fecal specimens (10/161, 6.21%) contrasted markedly with the high proportion of positive identification among submitted partial worms or worm fragments (94/96, 97.9%), highlighting the importance of morphological expertise in the identification of fragmented helminthic specimens. In contrast, all worm-like materials and insect larvae submitted by patients (74/74) were ultimately determined to be non-parasitic or unrelated to human helminthic infection.

### Demographic data and parasitic prevalence

According to data from the Medical Record Department, Hospital for Tropical Diseases, Faculty of Tropical Medicine, Mahidol University (2014–2022), among the 4,055 patient records, 868 patients with suspected parasitic infections sought care at the Parasite Excellence Clinic, comprising cases of parasitic infections, no parasitic infections (i.e., normal), and DP. Among the helminthic infections, gnathostomiasis (n = 366) and taeniasis (n = 245) were the most prevalent, followed by strongyloidiasis (n = 39), hookworm infection (n = 24), enterobiasis (n = 18), opisthorchiasis (n = 13), cysticercosis (n = 10), ascariasis and trichuriasis (both, n = 4), filariasis and fascioliasis (both, n = 3), paragonimiasis (n = 2), and angiostrongyliasis (n = 1). There were 13 cases classified as unspecified visceral larva migrans (Table 2).

**Table 2:** Summary of patients infected with parasitic infections from the Parasite Excellence Clinic, categorized by diagnosis, sex, and age range (based on outpatient department records).

Diagnosis	Total	Sex		Age	
		M	F	Range	Average
Angiostrongyliasis	1	1	0	29	29
Ascariasis	4	2	2	22-89	38.4
Cutaneous/Visceral larva migrant	13	5	8	17-42	38.68
Enterobiasis	18	7	11	8-80	41
Filariasis	3	3	0	34-86	59.1
Gnathostomiasis	366	133	233	7-89	47.9
Hookworm infection	24	13	11	20-67	40.6
Strongyloidiasis	39	30	9	18-79	37
Trichuriasis	4	3	1	42-73	57.3
Fascioliasis	3	2	1	22-44	33.7
Opisthorchiasis	13	5	8	24-76	45.1
Paragonimiasis	2	1	1	5-60	32.5
Cysticercosis	10	6	4	12-74	51.7
Taeniasis	245	174	71	7-79	40.48
Delusional parasitosis	84	33	51	24-89	55.8
Non-infection (Normal)^a^	39	19	20	11-89	37.6
**Total**	**868**	**437**	**431**		

^a^Normal: patients (i.e., not diagnosed with DP) who requested parasitic examination based on symptoms and/or observation of objects resembling parasites, where no parasites were detected in the laboratory examination.

**Fig 3:**
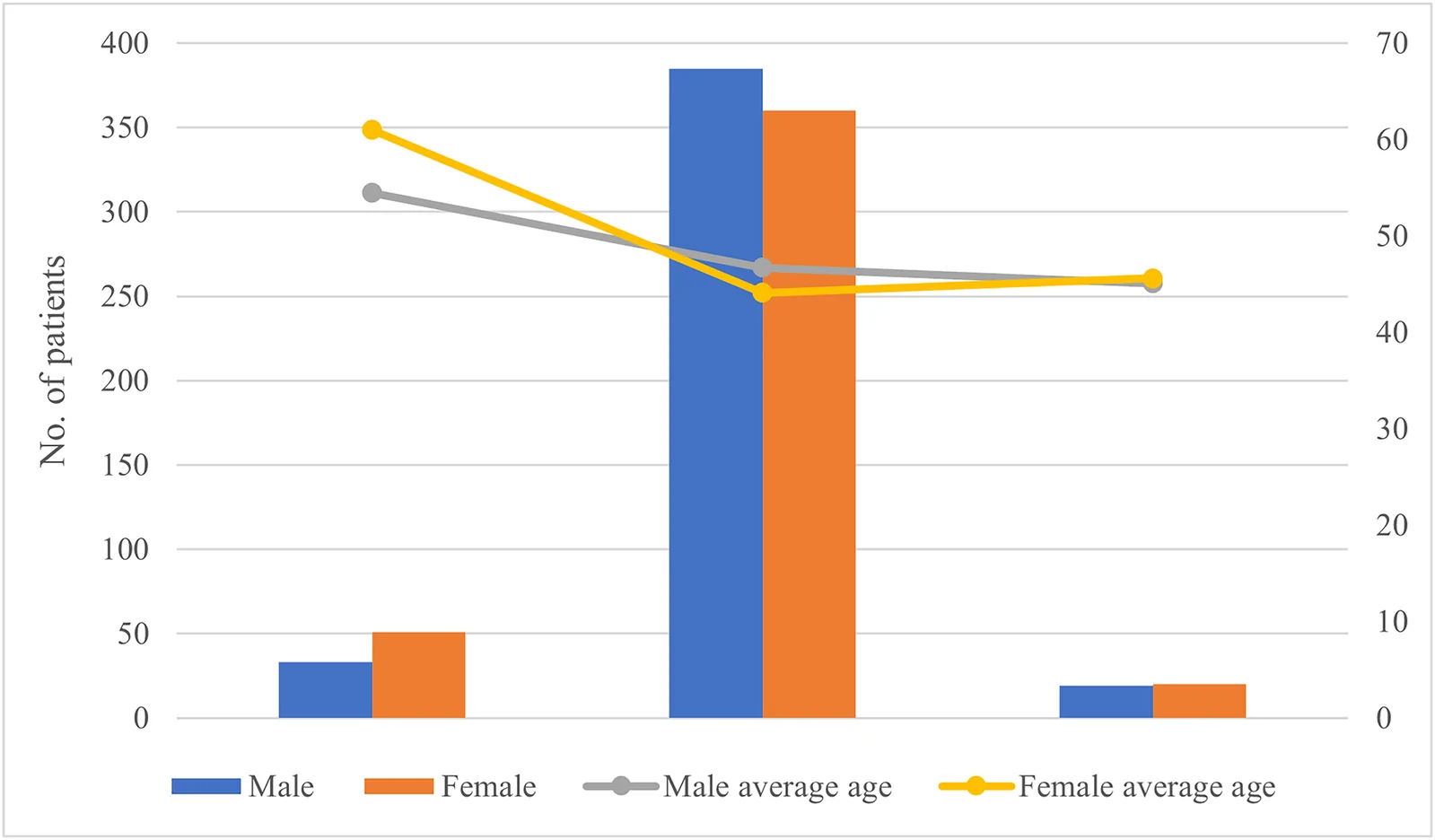
Graphical summary of parasitic diseases and their gender and age distribution.

The mean age distribution varied according to diagnosis. Gnathostomiasis was observed across a wide age range (7–89 years), with a mean age of ~48 years in both sexes. The mean age of patients with taeniasis was ~40 years, while opisthorchiasis tended to occur in older individuals, particularly males (mean age: 57.8 years). In contrast, the average age of patients with strongyloidiasis fascioliasis and paragonimiasis was younger. Patients with DP (n = 84) had the highest mean age (males: 54.5 years; females: 61.0 years), with a range of 22–91 years. Notably, compared with patients with confirmed parasitic infections, individuals diagnosed with DP tended to be older and were more commonly female. In addition, DP patients more frequently submitted non-biological materials, environmental debris, fibers, or skin-derived specimens rather than intact helminths or parasite eggs. Figure 3 presents a graphical summary of the disease, sex, and age data shown in Table 2.

Diagnostic methods included a combination of clinical history, microscopic identification, and immunological techniques. Fecal examinations and immunological tests were negative for 123 patients who presented with symptoms, some of whom reported observing objects resembling parasites. Among these patients, 39 were considered normal, and 84 were diagnosed with DP. Some patients had visited the Parasite Excellence Clinic several times and tested negative for parasitosis, while specimens from other patients were submitted to our laboratory from other hospitals or private laboratories.

## Discussion

**Fig 4:**
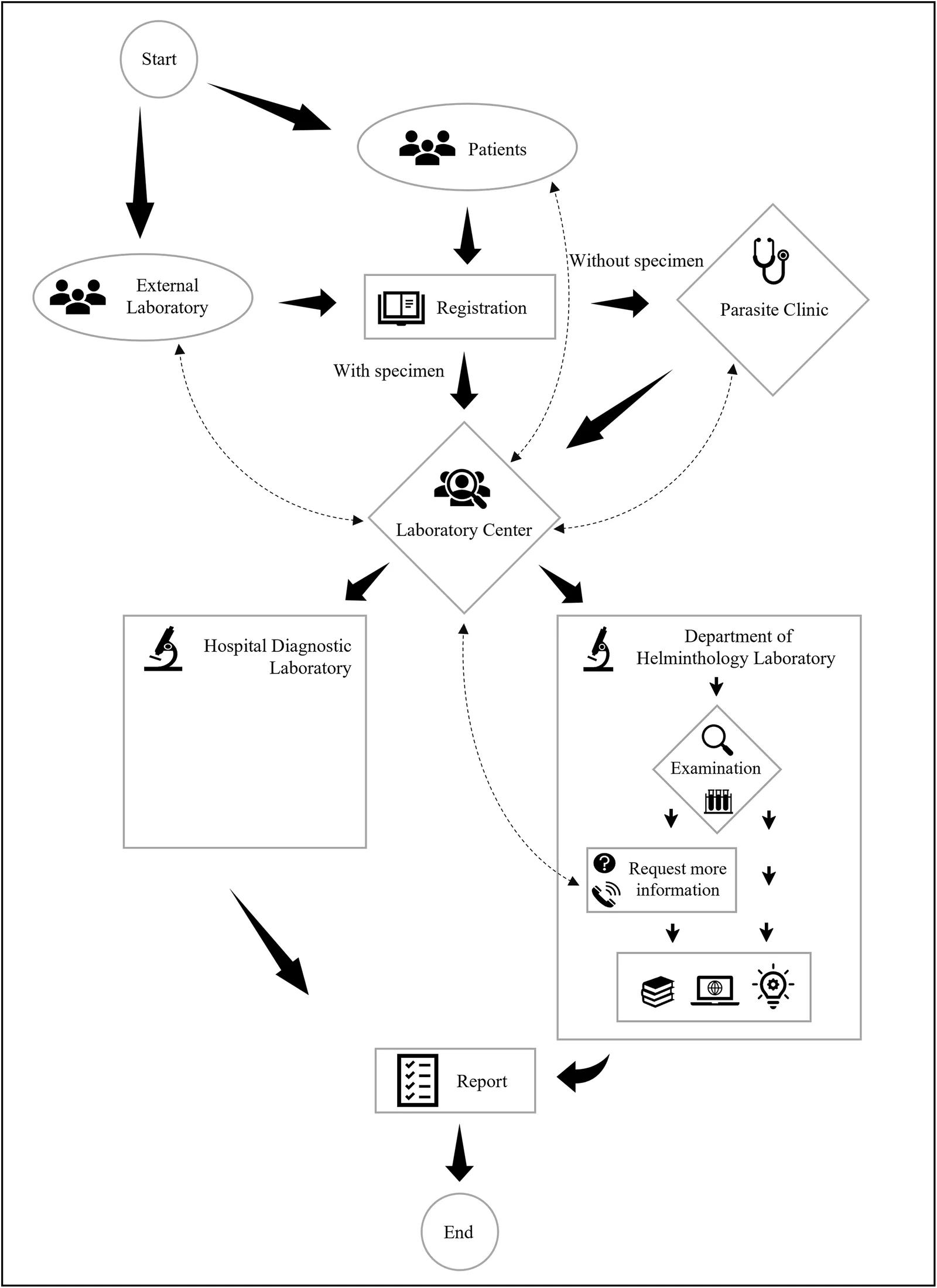
Diagnostic workflow, highlighting the role of the Department of Helminthology Laboratory, Faculty of Tropical Medicine, Mahidol University.

The longitudinal pattern observed throughout the study period highlights the evolving role of specialized parasitological laboratories in the assessment of patients with suspected parasitic infections. Although genuine parasitic infections were confirmed in a subset of cases, the majority of submitted specimens consisted of non-parasitic materials, emphasizing the diagnostic challenge posed by pseudoparasitic interpretations. Repeated specimen submission and persistent requests for parasitological confirmation among some patients are consistent with previously described healthcare-seeking behaviors in individuals with DP. Medical parasitologists play a critical role in differentiating real infections from false beliefs and gently guiding patients toward mental health support. Figure 4 presents the diagnostic workflow at the Department of Helminthology, Faculty of Tropical Medicine, Mahidol University.

Our findings highlight the dual burden of confirmed helminthic infections and DP in this hospital-based population. Gnathostomiasis and taeniasis remained the leading parasitic diseases, consistent with dietary risk factors, such as the consumption of raw or undercooked freshwater fish, eels [16], or pork or beef products [17], thereby facilitating transmission. The wide age distribution of patients with gnathostomiasis emphasizes that exposure and risk occur across all ages, while the higher mean age of patients with opisthorchiasis aligns with cumulative exposure to liver flukes in endemic regions. In several cases, the same patients submitted multiple samples that all tested negative for parasites.

The ability to distinguish true parasites from artifacts is essential in tropical diagnostics. Misidentification may lead to unnecessary antiparasitic treatment, psychological distress, and even iatrogenic complications. In our study, commonly misidentified materials included undigested food, insect larvae, and synthetic fibers, which, under low magnification, may resemble helminthic eggs or worm body parts, an observation consistent with studies on diagnostic errors in parasitology [18–20]. Specimens containing partial fragments of worms should be examined microscopically to determine whether they are true nematodes, cestodes, or trematodes. This requires accurate identification of the morphological features specific to each group, including the presence or absence of segmentation, the shape and structure of the cuticle, and internal anatomical features [21]. Due to their size and structure, stereomicroscopy may help with identification. Although *Anisakis* larvae have been reported in Thai marine fish, anisakiasis is not considered endemic in Thailand [22]. The four specimens in our study containing *Anisakis* larvae had been endoscopically collected from the gastrointestinal tract of a Japanese patient. In the first three specimens, a characteristic mucron was observed at the posterior end of *Anisakis* larvae. In the last specimen, although the mucron was not clearly identified, the morphological appearance, size, and color were sufficient for recognition [23]. Furthermore, the fact that the patient was from Japan, where *Anisakis* is considered endemic, supports the likelihood of this infection. Since juvenile earthworms do not have a clitellum, a multifunctional organ for reproduction and regeneration [24], they can be mistaken for juvenile *A. lumbricoides*. One of the specimens received in our study resembled an *Ascaris* worm due to its worm-like appearance and dark brown color. However, the absence of three lips and the presence of eye-like dots and scaled skin confirmed that it was a common blind snake [25]. These cases underscore the importance of careful morphological examination remained highly effective even for fragmented specimens to avoid misidentification of worm-like specimens.

Some specimens were submitted due to parasite-related concerns, particularly when individuals suspected they had encountered parasitic organisms. For instance, a practical nurse student who had recently studied parasitology submitted a worm-like specimen found in a flush toilet, suspecting a parasite. Stereomicroscopic examination revealed that the specimen was a midge larva, commonly found in aquatic sediments and likely introduced through household water contamination from a leaking pipeline [26]. This case highlights the importance of accurate identification in distinguishing true parasitic infections from environmental contaminants, emphasizing the value of providing knowledge and reassurance to those submitting such specimens.

Specimen quality is a crucial factor for accurate parasitic identification. When preserved in a suitable solution (e.g., 10% formalin or 70%–95% alcohol), parasites remain fresh and are easy to identify. If these solutions are unavailable, 0.85% normal saline at 4°C can be used for short-term storage and transport before examination [21]. Specimens kept in water may degrade and become unidentifiable. Moreover, the volume of preservative solution should be proportional to the container size, and insufficient solution during transportation may result in small specimens adhering to the lid or drying out, making identification difficult.

Another important factor for contributing to accurate parasitic identification is the availability of detailed clinical and specimen-related information. Documentation regarding the site of specimen collection, environmental source, duration of symptoms, travel history, dietary consumption, animal contact, prior antiparasitic treatment, and the specific reason for submission can substantially narrow the differential diagnosis and guide appropriate examination methods. Proper handling of fecal specimens is also essential and specimens should not be contaminated with urine. For example, specimens collected from bathrooms or toilet water may suggest environmental contamination or free-living organisms rather than true human parasitic infection. The detection of *Strongyloides* larvae requires a fresh fecal specimen stored and transported at room temperature and not refrigerated [27], as refrigeration may cause the larvae to become nonmotile or die, leading to a false-negative result. Similarly, knowledge of recent consumption of raw freshwater fish, undercooked meat, or travel to endemic regions may assist in prioritizing specific parasitic diagnoses. Standardized specimen submission forms and closer communication between clinicians and laboratory personnel may; therefore, improve diagnostic efficiency, reduce misinterpretation, and facilitate more accurate differentiation between true parasitic infection and nonparasitic materials in patients with suspected DP.

A substantial number of specimens, particularly those repeatedly submitted by the same individuals, were associated with complaints of itchy skin. In many such cases, the submitted materials were misinterpreted as parasites because of the patients’ heightened vigilance and concern about their symptoms. Anxiety and perceptual distortions appear to be responsible for the continued submission of nonparasitic materials, as noted in previous reports on the psychological aspects of perceived parasitic infections [10,22,28]. DP remains a complex clinical entity requiring interdisciplinary management. This psychotic disorder is characterized by a fixed, false belief of being infected with parasites or other organisms [3]. Patients frequently report itching, crawling sensations (formication), and visible “parasites” emerging from the skin. They often present to tropical medicine, dermatology, or parasitology clinics, bringing samples such as scabs, skin flakes, hair, or dust as “proof,” a behavior described as the matchbox sign [29]. Patients with DP are usually convinced that their symptoms are caused by living organisms. Ignoring or dismissing their claims exacerbates distress, erodes trust, and leads to doctor shopping or self-harm [30,31].

Patients with DP frequently seek repeated diagnostic confirmation. In Thailand, many patients with DP visit the Parasite Excellence Clinic at the Hospital for Tropical Diseases. Physicians routinely accept their specimens despite the absence of a true parasitic infections and submit them for laboratory examination. Submissions may also be received through external laboratories.

For laboratory technicians, repeatedly encountering clearly nonparasitic materials can become a tedious routine and, at times, unintentionally humorous. Although the identification of such materials does not typically require advanced tools, clinicians often assure patients that their samples have been examined using modern diagnostic equipment, such as high-magnification microscopes. This approach reinforces the credibility of the results by emphasizing that they come from a specialized parasitology laboratory rather than relying solely on clinical judgment. It also allows clinicians to deliver findings in a respectful, nonconfrontational manner, preserving the patient’s dignity and enhancing trust in the diagnostic process. Laboratory confirmation, particularly of negative results, has a profound therapeutic function, and patients with DP often refuse psychiatric referral until they are utterly convinced that no infection exists [3]. The emphatic delivery of a clear, authoritative report from both the laboratory and clinician helps patients with DP accept that the medical investigations were comprehensive and that alternative consultations, unnecessary antiparasitic treatments, and self-inflicted injuries from attempts to extract perceived parasites [10,29].

From a diagnostic standpoint, it is ethically and clinically mandatory to exclude genuine infection before considering a psychiatric diagnosis [32,33]. Several dermatologic and infectious conditions, including cutaneous larva migrants, strongylidiasis, gnathostomiasis [10,34], lice and flea mimic the sensations or lesions described in DP [3]. Thus, failure to identify a true parasitic infection may prolong patient suffering and lead to a loss of confidence in medical care.

Engaging with patients suffering from DP requires both skill and sensitivity. Clinicians must delicately navigate the patient’s firm belief in infection, which may involve pointing to skin lesions or presenting various self-collected materials, such as debris from the body or nearby surroundings, as alleged evidence of parasites. Rather than outright dismissal of these concerns, clinicians are encouraged to accept and formally submit such specimens for laboratory investigation. An official laboratory report indicating the absence of parasites is often more acceptable to patients than verbal reassurance. Without this validation, patients may continue seeking second opinions at other facilities, thereby prolonging their psychological distress and delaying appropriate intervention. Thus, building a therapeutic alliance is essential, and clinicians should patiently listen, express empathy, and acknowledge the suffering associated with the condition. Such rapport not only fosters trust but also opens the door for introducing psychiatric assessment and treatment, which are often initially resisted by patients [10].

A more formal integration of psychiatric evaluation into the diagnostic workflow would substantially improve the management of patients with suspected DP. In clinical practice, patients frequently undergo repeated parasitological examinations before psychiatric referral is considered, often resulting in prolonged diagnostic trajectories and unnecessary antiparasitic treatment. Practical implementation strategies may include standardized referral criteria after repeated negative parasitological investigations, multidisciplinary case conferences involving parasitologists, dermatologists, psychiatrists, and infectious disease specialists, and the incorporation of brief psychiatric screening tools into routine clinical assessment. Establishing dedicated communication channels between clinicians and laboratory personnel is also important, as laboratory findings frequently influence patient acceptance of subsequent psychiatric referral. Regular interdisciplinary meetings and shared case discussions may improve mutual understanding of DP and facilitate earlier recognition of psychocutaneous disorders. Furthermore, maintaining empathetic, nonconfrontational communication with patients remains essential for preserving trust and encouraging acceptance of psychiatric evaluation and treatment. Such an integrated model may reduce repeated healthcare utilization, unnecessary laboratory investigations, inappropriate antiparasitic treatment, and self-inflicted skin injury associated with persistent attempts to remove perceived parasites. Continuous communication between clinical and laboratory teams has proven valuable. Through regular interaction with attending clinicians, laboratory technicians have become more aware of the clinical features and significance of DP, a condition that was previously unfamiliar to many. This exchange has enhanced interdisciplinary understanding, helping both clinicians and laboratory technicians to better appreciate the complexity of DP and the patient experience. Furthermore, because DP is not well-known to generalists, the condition often goes undiagnosed or is incorrectly diagnosed. Patients with DPs usually visit numerous professionals, such as physicians, medical parasitologists, hygienists, and entomologists, for treatment, and have been suffering from their condition for a long time. Once patients with DP are informed of negative findings or any that deviate from their perceptions of infection, they express consistent and fierce rejection.

There were 84 patients in our study who met the definition of DP, believing that their body surfaces were infected with insects or parasites, despite clinicians being unable to find confirmatory evidence, consistent with negative laboratory results and confirmation that suspected pathogenic organisms were, in fact, skin debris. Some patients showed excoriation due to scratching. In our study, DP prevalence was higher among older females, consistent with a meta-analysis of 1,223 case reports over 100 years, which found that most cases occurred in middle-aged and older women with a high socioeconomic status [2].

In the context of DP, dermatologists and medical parasitologists often serve as the first point of contact, and their role in patient communication, differential diagnosis, and guidance toward appropriate psychiatric care is critical. Presenting symptoms include self-mutilation, ranging from scratches to deep ulceration at sites where the patients have attempted to extricate the causative organisms. Self-inflicted lesions in patients with DP have been observed after hours of attempting to remove either worms or insects from under the skin using tweezers [28].

Microscopy remains fundamental in parasitological diagnosis, particularly in resource-limited settings. Although molecular diagnostics offer improved specificity and sensitivity, this approach may be unfeasible due to cost and infrastructure constraints [34]. Therefore, basic training in morphological parasite identification remains an essential skill for parasitologists and laboratory technicians. Furthermore, collecting detailed information from patients who submit specimens, such as the collection site and the reason for submission, helps narrow the scope of identification, facilitating accurate analysis. In settings where access to psychiatric care is limited or stigmatized, parasitological laboratories may serve as vital entry points, not only for ruling out parasitic infections but also for initiating dialog that may eventually lead to psychiatric evaluation. This dual role underscores the importance of diagnostic discernment and training in compassionate communication, particularly when managing patients with suspected DP. Treatment is usually accepted if the threatening etiology is identified, followed by the prescription of appropriate medication. In cases where laboratory investigations have not been performed, it is relatively difficult to convince patients to receive psychiatric medication.

### Limitations

This study has some limitations. First, many externally submitted specimens were accompanied by minimal or no clinical history, limiting the ability to correlate laboratory findings with patient symptoms or outcomes. Second, the lack of standardized follow-up protocols impeded confirmation of clinical diagnoses, particularly in cases of suspected DP. The absence of structured psychiatric assessments or longitudinal clinical data made it difficult to verify psychological conditions or evaluate patient responses to referrals and treatment. Third, because of the retrospective nature of the study and incomplete documentation of prior consultations outside the Hospital for Tropical Diseases, accurate calculation of the interval between initial presentation and final diagnosis of DP was not possible for all patients. Additionally, molecular confirmation was not performed for ambiguous samples, potentially limiting diagnostic resolution in cases where morphological identification was inconclusive. These limitations highlight the need for integrated clinical, laboratory, and psychiatric collaboration for managing patients presenting with suspected parasitic infections. Future prospective studies should incorporate standardized psychiatric assessments, structured longitudinal follow-up, and multidisciplinary evaluation protocols to better characterize the psychological profiles, treatment responses, healthcare-seeking behaviors, and diagnostic trajectories of patients with suspected DP. Furthermore, formal integration of psychiatric services into tropical medicine and parasitology clinics may improve diagnostic accuracy, facilitate earlier intervention, and reduce delays in appropriate psychiatric referral and treatment.

## Conclusions

Accurate and well-documented laboratory confirmation is the cornerstone of diagnostic and therapeutic management in suspected DP. Microscopy plays an indispensable role in identifying parasitic infections and distinguishing them from artifacts is critical not only for guiding appropriate antiparasitic treatment but also for preventing misdiagnosis and facilitating the recognition of psychiatric conditions. Strengthening collaboration between clinicians and diagnostic laboratories, alongside raising DP awareness, will significantly improve patient outcomes. A collaborative framework, where parasitologists objectively exclude infection and clinicians communicate results compassionately, represents best practice in terms of patient safety and quality of care.
